# Flexible and efficient Bayesian pharmacometrics modeling using Stan and Torsten, Part I

**DOI:** 10.1002/psp4.12812

**Published:** 2022-06-23

**Authors:** Charles C. Margossian, Yi Zhang, William R. Gillespie

**Affiliations:** ^1^ Department of Statistics Columbia University (formerly Metrum Research Group, Inc.) New York New York USA; ^2^ Metrum Research Group, Inc. Tariffville Connecticut USA

## Abstract

Stan is an open‐source probabilistic programing language, primarily designed to do Bayesian data analysis. Its main inference algorithm is an adaptive Hamiltonian Monte Carlo sampler, supported by state‐of‐the‐art gradient computation. Stan's strengths include efficient computation, an expressive language that offers a great deal of flexibility, and numerous diagnostics that allow modelers to check whether the inference is reliable. Torsten extends Stan with a suite of functions that facilitate the specification of pharmacokinetic and pharmacodynamic models and makes it straightforward to specify a clinical event schedule. Part I of this tutorial demonstrates how to build, fit, and criticize standard pharmacokinetic and pharmacodynamic models using Stan and Torsten.

## INTRODUCTION

Bayesian inference offers a principled approach to learn about unknown variables from data using a probabilistic analysis. The conclusions we draw are based on the posterior distribution that, in all but the simplest cases, is intractable. We can, however, probe the posterior using a host of techniques such as Markov Chain Monte Carlo (MCMC) sampling and approximate Bayesian computation. Writing these algorithms is a tedious and error‐prone endeavor but fortunately modelers can often rely on existing software with efficient implementations.

In the field of pharmacometrics, statistical software such as NONMEM®,^1^ Monolix®,^2^ and the R package nlmixr^3^ support many routines to specify and analyze pharmacokinetic (PK) and pharmacodynamic (PD) population models. There also exist more general probabilistic programing languages such as BUGS^4^ and more recently Stan,^5^ to name only a few examples. This tutorial focuses on Stan. Stan supports a rich library of probability densities, mathematical functions including matrix operations, and numerical solvers for differential equations. These features make for an expressive and flexible language; however, writing common pharmacometrics models can be tedious. Torsten extends Stan by providing a suite of functions to facilitate the specification of pharmacometrics models. These functions make it straightforward to model the event schedule of a clinical trial and parallelize computation across patients for population models.

This tutorial reviews key elements of a Bayesian modeling workflow in Stan, including model implementation, inference using MCMC, and diagnostics to assess the quality of our inference and modeling. We assume the reader is familiar with compartment models in PK and PD and has experience with data that describe a clinical event schedule. Because Torsten follows the input conventions in NMTRAN®, experience with NONMEM® is helpful although not essential. Likewise, exposure to Bayesian statistics and inference algorithms is desirable, in particular an elementary understanding of MCMC.

We introduce programming in Stan and Torsten with the assumption that the reader is familiar with R.

### Why Stan?

We believe that Stan, coupled with Torsten, can be an important addition to the pharmacometrician's toolkit, especially for Bayesian data analysis.

The most obvious strength of Stan is its flexibility: it is straightforward to specify priors, systems of ordinary differential equations (ODEs), a broad range of measurement models, missing data models and complex hierarchies (i.e., population models). Examples of how Stan's flexibility may be leveraged in pharmacometrics include the following:

- Combining various sources of data and their corresponding measurement models into one large model, over which full Bayesian inference can be performed (e.g., Weber et al.^6^). In a similar vein, it is possible to build complex hierarchical structures that allow us to simultaneously pool information across various groups, for example, patients, trials, or countries. We will study such an example in Part 2 of this tutorial.
- Using a sparsity inducing prior, such as a the *Horseshoe* prior,^7^, ^8^ to fit models with a high‐dimensional covariate. This approach has, for example, been used in oncology^9^ and is a promising avenue in pharmacogenetics.^10^
- Incorporating a non‐parametric regression, such as a Gaussian process, to build a translational model for pediatric studies (e.g., Siivola et al.^11^).

Stan's expressive language plays a crucial part here because more specialized software do not readily handle the relatively complex structures and priors the previous examples require.

In addition, Stan supports state‐of‐the‐art inference algorithms, most notably an adaptive Hamiltonian Monte Carlo (HMC) sampler, a gradient‐based MCMC algorithm^12^ based on the No U‐Turn sampler (NUTS),^13^ automatic differentiation variational inference (ADVI),^14^ and penalized maximum likelihood estimators. Stan's inference algorithms are supported by a modern automatic differentiation library that efficiently generates the requisite derivatives.^15^ It is worth pointing out that algorithms such as NUTS and ADVI were first developed and implemented in Stan before being widely adopted by the applied statistics and modeling communities. As of the writing of this article, new inference algorithms continue to be prototyped in Stan. Recent such examples include adjoint‐differentiated Laplace approximations,^16^ cross‐chain warmup,^17^ and path finding for improved chain initialization.^18^ Some of Stan's algorithms are now available in specialized pharmacometrics software. NONMEM® supports an HMC sampler, although certain diagnostics required to assess the quality of HMC, notably for population models, are still missing.

Stan indeed provides a rich set of diagnostics, including the detection of divergent transitions during HMC sampling,^12^ and the improved computation of effective sample sizes and scale reduction factors, $\hat{R}$_,_ ^19^ as well as detailed warning messages based on these diagnostics. The automated running of these diagnostics makes the platform more user friendly and provides much guidance when troubleshooting our model and our inference.

Last but not least, both Stan and Torsten are open‐source projects, meaning they are free and their source code can be examined and, if needed, scrutinized. The projects are under active development with new features being added regularly.

### Bayesian inference: notation, goals, and comments

Given the observed data D and latent variables θ from the parameter space Θ, a Bayesian model is defined by the joint distribution $p\left(D,\theta \right)$. The latent variables can include model parameters, missing data, and more. In this tutorial, we are mostly concerned with estimating model parameters.

The joint distribution observes a convenient decomposition,

$$p\left(D,\theta \right)=p\left(\theta \right)p\left(D|\theta \right),$$

with $p\left(\theta \right)$ the *prior* distribution and $p\left(D|\theta \right)$ the *likelihood*. The prior encodes information about the parameters, usually based on scientific expertise or results from previous analysis. The likelihood tells us how the data are distributed for a fixed parameter value and, per one interpretation, can be thought of as a “story of how the data is generated.”^20^ The Bayesian proposition is to base our inference on the *posterior* distribution of the parameters, $p\left(\theta |D\right)$, and more generally the posterior distribution of any derived quantity of interest, $p\left(f\left(\theta \right)|D\right)$.

For typical pharmacometric applications, the full joint posterior density of the model parameters is an unfathomable object that lives in a high‐dimensional space. Usually we cannot even numerically evaluate the posterior density at any particular point! Instead, we must probe the posterior distribution and learn the characteristics that interest us the most. In our experience, this often includes a measure of a central tendency and a quantification of uncertainty, for example, the mean and the variance, or the median and the 5th and 95th quantiles for any quantity of interest. For skewed or multimodal distributions, we may want a more refined analysis that looks at many quantiles. What we compute are estimates of these quantities. Most Bayesian inference involves calculations based on marginal posterior distributions. That typically requires integration over a high number of dimensions—an integration that is rarely tractable by analytic or numerical quadrature. One strategy is to generate approximate samples from the posterior distribution and then use the sample mean, sample variance, and sample quantiles as our estimators.

Bayes' rule teaches us that

$$p\left(\theta |D\right)=\frac{p\left(D,\theta \right)}{p\left(D\right)}=\frac{p\left(D|\theta \right)p\left(\theta \right)}{p\left(D\right)}.$$

Typically we can evaluate the joint density in the numerator but not the normalizing constant, $p\left(D\right)$, in the denominator. A useful method must therefore be able to generate samples from the posterior $p\left(\theta |D\right)$ using the *unnormalized* posterior density, $p\left(D,,,\theta \right)$. Once we generate a sample θ, we can apply a transformation f to obtain a sample from $p\left(f\left(\theta \right)|D\right)$.

Many MCMC algorithms are designed to generate samples from an unnormalized density. Starting at an initial point, these chains explore the parameter space Θ, one iteration at a time, to produce the desired samples. The first iterations of MCMC are used to find and explore the region in the parameter space where the posterior probability mass concentrates. Only after this initial *warmup phase* do we begin the *sampling phase*.

HMC is an MCMC method that uses the gradient to efficiently move across the parameter space.^12^, ^21^ Computationally, running HMC requires evaluating $\log\;p\left(D,\theta \right)$ and $\nabla_{\theta }\log\;p\left(D,\theta \right)$ many times across Θ, that is, for varying values of θ but fixed values of D. For this procedure to be well defined, θ must be a continuous variable, else the requisite gradient does not exist. Discrete parameters require a special treatment, which we will not discuss in this tutorial.

A Stan program specifies a method to evaluate $\log\;p\left(D,\theta \right)$. Thanks to automatic differentiation, this implicitly defines a procedure to compute $\nabla_{\theta }\log\;p\left(D,\theta \right)$_._ ^22^, ^23^, ^24^ Together, these two objects provide all the relevant information about our model to run HMC sampling and other gradient‐based inference algorithms.

### Bayesian workflow

Bayesian inference is only one step of a broader modeling process, which we might call the Bayesian workflow.^12^, ^25^, ^26^ Once we fit the model, we need to check the inference and, if needed, fine tune our algorithm, or potentially change method. And once we trust the inference, we naturally need to check the fitted model. Our goal is to understand the shortcomings of our model and motivate useful revisions. During the early stages of model development, this mostly comes down to troubleshooting our implementation, and later this “criticism” step can lead to deeper insights.

All through the tutorial, we demonstrate how Stan and Torsten can be used to check our inference and our fitted model.

### Setting up Stan and Torsten

Detailed instructions on installing Stan and Torsten can be found on https://github.com/metrumresearchgroup/Torsten. At its core, Stan is a C++ library, but it can be interfaced with one of many scripting languages, including R, Python, and Julia. Running Stan requires a modern C++ compiler such as g++ 8.1 provided by RTools 4.0 on Windows and the GNU‐Make utility program on Mac or the Windows equivalent mingw32‐make. More details of setting up work environment can be found in the CmdStan User's Guide.^27^ We will use cmdStanR, which is a lightweight wrapper of Stan in R, and in addition, the packages posterior,^28^ bayesplot,^29^ and loo.^30^ We generate most of the figures in this article using BayesPlot, although at times we trade convenience for flexibility and fall back to ggplot2.^31^

The R and Stan code for all examples are available at https://github.com/metrumresearchgroup/torsten_tutorial_1_supplementary.

### Resources

Helpful reads include the *Stan User Manual* ^32^ and the *Torsten User Manual*.^33^ *Statistical Rethinking* by McElreath^34^ provides an excellent tutorial on Bayesian analysis that may be used for self‐learning. A comprehensive textbook on Bayesian modeling is *Bayesian Data Analysis* by Gelman et al.,^20^ with more recent insights on the Bayesian workflow provided by Gelman et al.^26^ Betancourt^12^ offers an accessible discussion on MCMC methods with an emphasis on HMC.

## TWO‐COMPARTMENT MODEL

As a starting example, we demonstrate the analysis of longitudinal plasma drug concentration data from a single individual using a linear two‐compartment model with first‐order absorption. The individual receives multiple doses at regular time intervals, and the plasma drug concentration is recorded over time. Our goal is to estimate the posterior distribution of the parameters of the model describing the time course of the plasma drug concentrations in this individual.

### PK model and clinical event schedule

Let us assume an individual receives a drug treatment of 1200 mg boluses q12h × 14 doses. Drug concentrations are measured in plasma obtained from blood sampled at 0.083, 0.167, 0.25, 0.5, 0.75, 1, 1.5, 2, 3, 4, 6, and 8 hours following the first, second, and final doses. In addition, we take measurements before each drug intake as well as 12, 18, and 24 h following the last dose. We analyze that data using a two‐compartment model with first‐order absorption:

(1a) $$\frac{du_{\mathrm{gut}}}{dt}=-k_{a}u_{\mathrm{gut}}$$

(1b) $$\frac{du_{\text{cent}}}{dt}=k_{a}u_{\mathrm{gut}}-\left(\frac{\mathrm{CL}}{V_{\text{cent}}}+\frac{Q}{V_{\text{cent}}}\right)u_{\text{cent}}+\frac{Q}{V_{\text{peri}}}u_{\text{peri}}$$

(1c) $$\frac{du_{\text{peri}}}{dt}=\frac{Q}{V_{\text{cent}}}u_{\text{cent}}-\frac{Q}{V_{\text{peri}}}u_{\text{peri}}$$

with

• $u\left(t\right)$: drug amount in each compartment (mg),

• $k_{a}$: absorption rate constant (h^−1^),

• CL: elimination clearance from the central compartment (L/h),

• Q: intercompartmental clearance (L/h),

• $V_{\text{cent}}$: volume of the central compartment (L),

• $V_{\text{peri}}$: volume of the peripheral compartment (L).

Both intervention and measurement events are described by the event schedule. Stan does not have any reserved variable names, but in this tutorial, we follow the NONMEM convention to specify events using the variable names in Table 1. More details can be found in the *Torsten User Manual*.

**TABLE 1:** Variables used specify an event schedule

Variable	Description
cmt	Compartment in which event occurs
evid	Type of event: (0) measurement, (1) dosing
addl	For dosing events, number of additional doses
ss	Steady state indicator: (0) no, (1) yes
amt	Amount of drug administered
time	Time of the event
rate	For dosing by infusion, rate of infusion
ii	For events with multiple dosing, interdose interval

### Statistical model

Given a treatment, x, and the PK parameters $\left\{k_{a},Q,\mathrm{CL},V_{\text{cent}},V_{\text{peri}}\right\}$, we compute the drug amounts u by solving the two‐compartment ODE. We use y to denote the measured drug concentration and $\hat{c}\left(=u/V_{\text{cent}}\right)$ the model‐predicted drug concentration. We model the residual error from y to $\hat{c}$ using a lognormal distribution

$$y|\hat{c},\sigma ∼\text{logNormal}\left(\log\;\hat{c},\sigma \right),$$

where σ is a scale parameter we wish to estimate. The deterministic computation of $\hat{c}$ along with the measurement model define our likelihood function $p\left(y|\theta ,x\right)$, where $\theta =\left\{k_{a},\mathrm{CL},Q,V_{\text{cent}},V_{\text{peri}},\sigma \right\}$ and x are input data, that is, the clinical event schedule. Note that we are not limited to the above simple model. Stan is capable of many distributions^35^ as well as encoding more complex residual models such as the proportional and additive error variance.

It remains to define a prior distribution, $p\left(\theta \right)$. Our prior should allocate probability mass to every plausible parameter value and exclude patently absurd values. For example, the volume of the central compartment is on the order of 10 L, but it cannot be the size of the sun. In this simulated example, our priors for the individual parameters are based on population estimates from previous (hypothetical) studies.

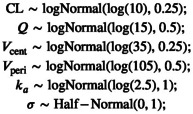

Suggestions for building priors can be found in Gabry et al.^25^ and Betancourt^36^ and at https://github.com/stan‐dev/stan/wiki/Prior‐Choice‐Recommendations.

### Specifying a model in Stan

We can now specify our statistical model using a Stan file, which is divided into coding blocks, each with a specific role. From R, we then run inference algorithms that take this Stan file as an input.

#### Data and parameters block

To define a model, we need a procedure that returns the log joint distribution, $\log\;p\left(D,\theta \right)$. Our first task is to declare the data, D, and the parameters, θ, using the coding blocks data and parameters. It is important to distinguish the two. The data are fixed. By contrast, the parameter values change as HMC explores the parameter space, and gradients of the joint density are computed with respect to θ, but not D.

For each variable we introduce, we must declare a type and, for containers such as arrays, vectors, and matrices, the size of the container (Chapter 5 in the Stan User's Guide^37^). In addition, each statement ends with a semicolon. It is possible to specify constraints on the parameters using the keywords lower and upper. If one of these constraints is violated, Stan returns an error message. More important, constrained parameters are transformed into unconstrained parameters—for instance, positive variables are put on the log scale—which greatly improves computation.

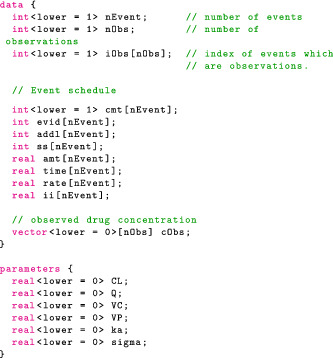

#### Model block

Next, the model block allows us to modify the variable target, which Stan recognizes as the log joint distribution. The following statement increments target using the prior on σ, which is a normal density, truncated at 0 to only put mass on positive values.

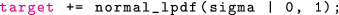

The truncation is implied by the fact σ is declared as lower bounded by 0 in the parameters block. An alternative syntax is the following:

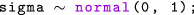

This statement now looks like our statistical formulation and makes the code more readable. We should be mindful that this is not a sampling statement but, rather, instructions on how to increment target. We now give the full model block:

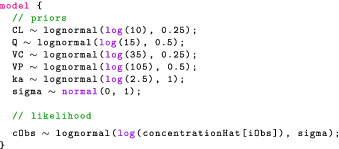

The likelihood statement involves a crucial term we have not defined yet: concentrationHat. Additional variables can be created using the transformed data and transformed parameters blocks. We will take advantage of these to compute the drug concentration in the central compartment for each event. Note that for the likelihood, we only use the concentration during observation events, hence the indexing [iObs].

#### Transformed data and transformed parameters block

In transformed data, we can construct variables that only depend on the data. For this model, we simply specify the number of compartments in our model (including the gut), nCmt, and the numbers of PK parameters, nTheta, two variables that will come in handy shortly.

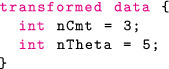

Because the data are fixed, this operation is only computed once. By contrast, operations in the transformed parameters block need to be performed (and differentiated) for each new parameter value.

To compute concentrationHat we need to solve the relevant ODE within the clinical event schedule. Torsten provides a function that returns the drug mass in each compartment at each timepoint of the event schedule.

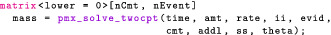

The first eight arguments define the event schedule and the last argument, theta, is an array containing the PK parameters, and defined as follows:

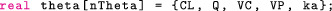

It is also possible to have theta change between events and specify lag times and bioavailability fractions, although we will not take advantage of these features in the example at hand.

The Torsten function we have chosen to use solves the ODEs analytically. Other routines use a matrix exponential, a numerical solver, or a combination of analytical and numerical methods.^38^ It now remains to compute the concentration in the central compartment at the relevant times. The full transformed parameters block is as follows:

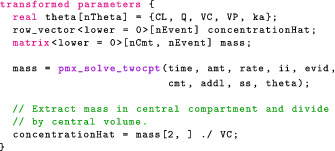

The Stan file contains all the coding blocks in the following order: data, transformed data, parameters, transformed parameters, model. The full Stan code can be found in the https://github.com/metrumresearchgroup/torsten_tutorial_1_supplementary.

### Calling Stan from R

The R package cmdstanr allows us to run a number of algorithms on a model defined in a Stan file. An excellent place to get started with the package is https://mc‐stan.org/cmdstanr/articles/cmdstanr.html.

The first step is to “transpile” the file—call it twocpt.stan—that is, translate the file into C++ and then compile it.

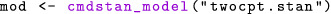

We can then run Stan's HMC sampler by passing in the requisite data and providing other tuning parameters; in this case the specified tuning parameters are (i) the number of Markov chains (which we run in parallel), (ii) the initial value for each chain, (iii) the number of warmup iterations, and (iv) the number of sampling iterations.

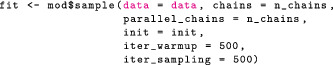

By default, Stan uses 1000 warmup iterations and 1000 sampling iterations. Empirically these defaults work well across a broad range of models when running an adaptive HMC sampler. For relatively simple models, we may even use shorter warmup and sampling phases, as we have done previously. This should be contrasted with random walk MCMC, such as the Gibbs sampler in BUGS, where it is typical to run 5000 or even 10,000 iterations per phase. Random walk MCMC tends to generate Markov chains with a higher autocorrelation than HMC, hence the need to run more iterations. In the next two sections, we discuss diagnostics that can be used to adjust the length of the warmup and sampling phases.

There are several other arguments we can pass to the sampler and that we will take advantage of throughout the tutorial. For applications in pharmacometrics, we recommend specifying the initial starting points via the init argument, as the defaults may not be appropriate. In this tutorial, we draw the initial points from their priors by defining an appropriate R function.

The resulting fit object stores the samples generated by HMC from which we can deduce the sample mean, sample variance, and sample quantiles of our posterior distribution. This information is readily accessible using fit$summary() and summarized in Table 2. We could also extract the samples and perform any number of operations on them.

**TABLE 2:** Summary of results when fitting a two‐compartment model

	Mean	Median	sd	mad	q5	q95	$\hat{R}$	ESS_bulk_	ESS_tail_
CL	10.0	10.0	0.378	0.367	9. 39	10.6	1.00	1580	1348
Q	19.8	19.5	4.00	4.01	13.8	26.8	1.00	985	1235
$V_{\text{cent}}$	41.2	40.8	9.71	9.96	25.6	57.7	1.00	732	1120
$V_{\text{peri}}$	124	123	18.0	18.0	97.1	155	1.00	1877	1279
$k_{a}$	1.73	1.67	0.523	0.522	1.01	2.68	1.00	762	1108
σ	0.224	0.222	0.0244	0.0232	0.187	0.269	1.01	1549	1083

*Note*: The first columns return sample estimates of the posterior mean, median, standard deviation, median absolute deviation, 5th and 95th quantiles, based on our approximate samples. The next three columns return the $\hat{R}$ statistics and the effective sample size for bulk and tail estimates, and can be used to identify problems with our inference.

### Checking our inference

Unfortunately there is no guarantee that a particular algorithm will work across all the applications we will encounter. We can, however, make sure that certain necessary conditions are met.

Much of the MCMC literature focuses on estimating expectation values for quantities of interest f,

$$Ef=\underset{\text{Θ}}{\int }f\left(\theta \right)p\left(\theta \text{|}y\right)\text{d}\theta ,$$

using sample estimators

$$\hat{E}f=\frac{1}{n}\sum_{i=1}^{n}f\left(\theta^{\left(i\right)}\right),$$

for some samples $\theta^{\left(1\right)},\theta^{\left(2\right)},\cdots ,\theta^{\left(n\right)}$. When constructing such estimators using MCMC samples, rather than with exact independent samples, we must account for the fact that our samples are correlated and biased.

#### Checking for convergence with $\hat{R}$

MCMC samples are biased because Markov chains generate correlated samples, meaning any sample has some correlation with the initial point. If we run the algorithm for enough iterations, the correlation to the initial point becomes negligible and the chain “forgets” its starting point. But what constitutes enough iterations?

To monitor bias, we run multiple Markov chains, each started at different points, and check that they all converge to the same region of the parameter space. One way to check this is to compute the $\hat{R}$ statistics, for which we provide an intuitive definition:

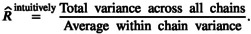

**FIGURE 1:**
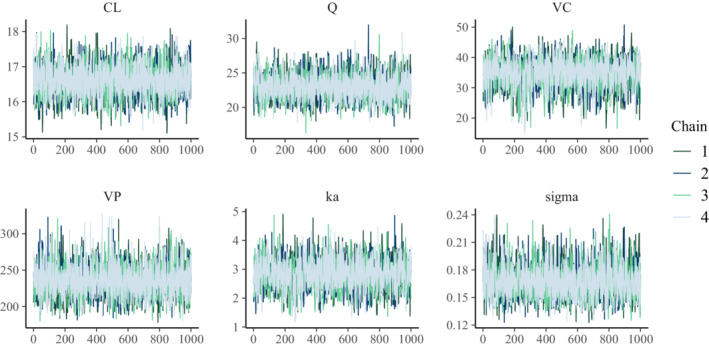
Trace plots. The sampled values for each parameters are plotted against the iterations during the sampling phase. Multiple Markov chains were initialized at different points. However, once in the sampling phase, we cannot distinguish the chains.

If the chains are mixing properly, both the numerator and denominator measure the posterior variance, and $\hat{R}$ converges to 1.0, as n increases. Moreover, we want $\hat{R}\approx 1.0$, as is the case in Table 2. Stan uses an improved $\hat{R}$ statistics described in a recent article by Vehtari et al.^19^ We can also visually check that the chains are properly mixing using a trace plot (Figure 1).

If $\hat{R}\gg 1$ and, more generally, if the chains were not mixing, this would be cause for concern and an invitation to adjust our inference method. One potential solution is to increase the warmup length. Even when $\hat{R}\approx 1$, we should entertain the possibility that all the chains suffer from the same bias.

#### Controlling the variance of our estimator

Let us assume that our warmup phase is long enough and the bias negligible. The expected error of our sample estimator is now determined by the variance. Under certain regularity conditions, our estimator follows an *MCMC central limit theorem*,

(2) $$\hat{E}f\overset{\text{approx}}{∼}\text{Normal}\left(Ef,\frac{\sigma_{f}}{\sqrt{n_{\mathrm{eff}}}}\right)$$

where $n_{\mathrm{eff}}$ is the *effective sample size*, denoted ${\mathrm{ESS}}_{\text{bulk}}$ in Table 2. Deviations from this approximation have order $O\left(1/n_{\mathrm{eff}}^{2}\right)$. In the limiting case where we generate independent samples, $n_{\mathrm{eff}}=n$; however, when samples exhibit correlation, $n_{\mathrm{eff}}<n$ and the variance of our sample estimator increases. For CL, we have 2000 samples, but the effective sample size is 1580 (Table 2). If $n_{\mathrm{eff}}$ is low, our estimator may not be precise enough, and we should increase the sampling phase to generate more samples.

Achieving $n_{\mathrm{eff}}\approx 100$ is, in our experience, usually sufficient in an applied setting. This means that the variance of the sample estimator is 1% that of the posterior, as can be seen from Equation (2). At this point, the uncertainty is dominated by the intrinsic posterior variance rather than the error in our inference procedure. If the effective sample size is below 100 for certain quantities, Stan issues a warning message.

The effective sample size is only formally defined in the context of estimators for expectation values. We may also be interested in tail quantities, such as extreme quantiles, which are more difficult to estimate and require many more samples to achieve a desired precision. Vehtari et al.^19^ propose a generalization of the effective sample size for such quantities and introduce the *tail effective sample size*. This is to be distinguished from the traditional effective sample size, henceforth the *bulk effective sample size*. Both quantities are reported by Stan.

### Checking the model: posterior predictive checks

Once we develop enough confidence in our inference, we still want to check our fitted model. There are many ways of doing this. We may look at the posterior distribution of an interpretable parameter and see if it suggests implausible values. Or we may evaluate the model's ability to perform a certain task, for example, classification or prediction, as is often done in machine learning. In practice, we find it useful to do posterior predictive checks (PPCs), that is, simulate data from the fitted model and compare the simulation to the observed data (Chapter 6 in Gelman et al.^39^). Mechanically, the procedure is straightforward:

1. Draw the parameters from their posterior, $\tilde{\theta }∼p\left(\theta ,|,y\right).$
2. Draw the predicted observations from the likelihood, conditional on the drawn parameters, $\tilde{y}∼p\left(y|\tilde{\theta }\right)$.

This amounts to drawing observations from their posterior distribution, that is, $\tilde{y}∼p\left(\tilde{y}|y\right)$. Both the uncertainty due to our estimation and the uncertainty due to our measurement model propagate to our predictions.

Stan provides a generated quantities block, which allows us to compute values, based on sampled parameters. In our two‐compartment model example, the following code draws predicted observations from the likelihood:

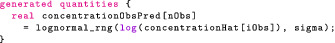

**FIGURE 2:**
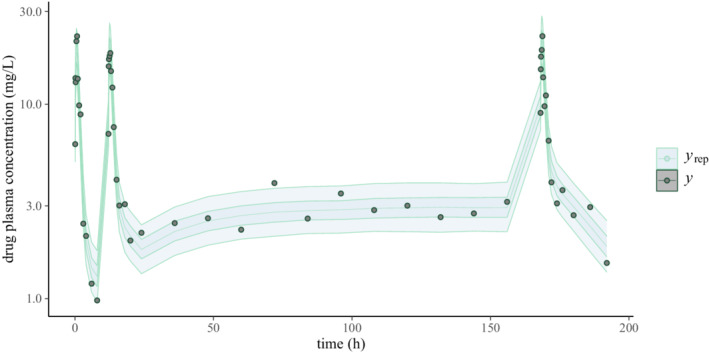
Posterior predictive checks for two‐compartment model. The circles represent the observed data (y) and the shaded areas the 50th and 90th credible intervals based on posterior draws ($y_{rep}$).

We generate predictions at the observed points for each sampled point, $\theta^{\left(i\right)}$. This gives us a sample of predictions, and we can use the 5th and 95th quantiles to construct a credible interval. We may then plot the observations and the credible intervals (Figure 2) and see that, indeed, the data generated by the model are consistent with the observations.

### Comparing models: leave‐one‐out cross‐validation

**FIGURE 3:**
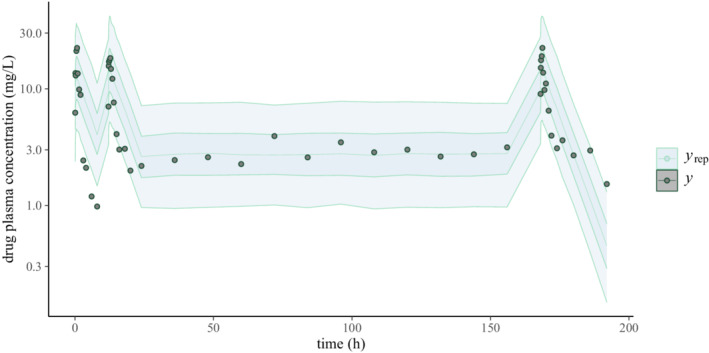
Posterior predictive checks for a one‐compartment model. The circles represent the observed data (y) and the shaded areas the 50th and 90th credible intervals based on posterior draws ($y_{rep}$). A graphical inspection suggests that the credible intervals are wider for the one‐compartment model than they are for the two‐compartment model.

Beyond model criticism, we may be interested in model comparison. Continuing our running example, we compare our two‐compartment model to a one‐compartment model, which is also supported by Torsten via the pmx_solve_onecpt routine. The corresponding PPCs are shown in Figure 3.

There are several ways of comparing models, and which method is appropriate crucially depends on the insights we wish to gain. If our goal is to assess a model's ability to make good out‐of‐sample predictions, we may consider *Bayesian leave‐one‐out* (LOO) cross‐validation. The premise of cross‐validation is to exclude a point, $\left(y_{i},,,x_{i}\right)$, from the *training set*, that is, the set of data to which we fit the model. Here, $x_{i}$ denotes the covariate, and in our example, the relevant row in the event schedule. We denote the reduced data set, $y_{-i}$. We then generate a prediction $\left({\tilde{y}}_{i},,,x_{i}\right)$ using the fitted model and compare ${\tilde{y}}_{i}$ to $y_{i}$. A classic metric to make this comparison is the squared error, ${\left({\tilde{y}}_{i}-y_{i}\right)}^{2}$.

Another approach is to use the *LOO estimate of out‐of‐sample predictive fit*:

$${\mathrm{elpd}}_{\mathrm{loo}}:=\sum_{i}^{n}\log\;p(y_{i}\;|\;y_{-i}).$$

Here, no prediction is made. Instead, we examine how consistent an “unobserved” data point is with our fitted model. Computing this estimator is expensive because it requires fitting the model to n different training sets in order to evaluate each term in the sum.

Vehtari et al.^40^ propose an estimator of ${\text{elpd}}_{\text{loo}}$, which uses Pareto smooth importance sampling and only requires a single model fit. The premise is to compute

$$\log\;p\left(y_{i}|y\right)$$

and correct this value, using importance sampling, to estimate $\log\;p\left(y_{i}|y_{-i}\right)$. Naturally this estimator may be inaccurate. What makes this tool so useful is that we can use the Pareto shape parameter, $\hat{k}$, to assess how reliable the estimate is. In particular, if $\hat{k}>0.7$, then the estimate should not be trusted. The estimator is implemented in the R package loo. See Gabry et al.^30^ for more details, including its connection and comparison to the widely applicable information criterion.

Conveniently, we can compute $\log\;p\left(y_{i}|y\right)$ in Stan's generated quantities block.

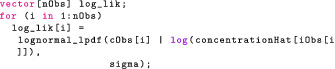

**FIGURE 4:**
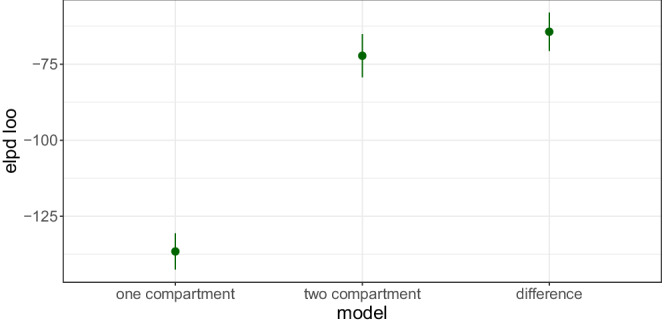
Leave‐one‐out estimate of out‐of‐sample predictive fit. Plotted is the estimate, ${\text{elpd}}_{\text{loo}}$, for the one‐ and two‐compartment models as well as the difference in ${\text{elpd}}_{\text{loo}}$ for the two models. Clearly, the two‐compartment model has superior predictive capabilities.

These results can then be extracted and fed into Loo to compute ${\text{elpd}}_{\text{loo}}$. The file twoCpt.r in https://github.com/metrumresearchgroup/torsten_tutorial_1_supplementary shows exactly how to do this. Figure 4 plots the estimated ${\text{elpd}}_{\text{loo}}$, along with a standard deviation, and shows the two‐compartment model has better out‐of‐sample predictive capabilities.

## TWO‐COMPARTMENT POPULATION MODEL

We now consider the scenario where we have data from multiple patients and fit a population model. Population models are a powerful tool to capture the heterogeneity between patients while recognizing similarities. Building the right prior allows us to pool information between patients, the idea being that what we learn from one patient teaches us something—although not everything—about the other patients. In practice, such models can frustrate inference algorithms and need to be implemented with care.^41^ We start with an example where the interaction between the model and our MCMC sampler is well behaved. In Part 2 of this tutorial, we examine a more difficult case for which we leverage Stan's diagnostic capabilities in order to run reliable inference.

### Statistical model

Let ϑ be the two‐dimensional array of body weight–normalized PK parameters for each patient, with

$$\begin{array}{c} \vartheta_{j}=\left({\mathrm{CL}}_{\mathrm{norm},\;j},Q_{\mathrm{norm},\;j},V_{\text{cent},\mathrm{norm},\;j},V_{\text{peri},\mathrm{norm},\;j},k_{a,\;j}\right), \end{array}$$

the parameters for the $j^{\mathrm{th}}$ patient. We construct a population model by introducing random variation to describe otherwise unexplained interindividual variability. In a Bayesian context, this is sometimes referred to as a prior distribution for the individual parameters,

$$\vartheta_{j}∼\text{LogNormal}\left(\log\;\vartheta_{\mathrm{pop}},\;\Omega \right).$$

As before, we work on the log scale to account for the fact the PK parameters are constrained to be positive. $\begin{array}{c} \vartheta_{\mathrm{pop}}=\left({\mathrm{CL}}_{\mathrm{pop}},\;Q_{\mathrm{pop}},\;V_{\text{cent},\mathrm{pop}},\;V_{\text{peri},\mathrm{pop}},\;k_{a,\mathrm{pop}}\right) \end{array}$ is the population mean (on the logarithmic scale) and Ω the population covariance matrix. Both $\vartheta_{\mathrm{pop}}$ and Ω are estimated. In this example, we start with the simple case where Ω is diagonal. For our example, we will also use conventional allometric scaling to adjust the clearance and volume parameters for body weight.

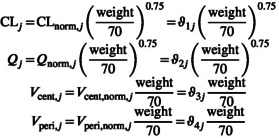

The likelihood remains mostly unchanged, with the caveat that it must now be computed for each patient. Putting this all together, we have the following model, as specified by the joint distribution,

$$\begin{array}{c} \begin{array}{l} \;\vartheta_{\mathrm{pop}}\;∼\;p\left(\vartheta_{\mathrm{pop}}\right),\;\left(\text{prior}\;\mathrm{on}\;\text{pharmacokinetic parameters}\right)\\ \;\Omega \;∼\;p\left(\Omega \right),\;\left(\text{prior}\;\mathrm{on}\;\text{population covariance}\right)\\ \;\sigma \;∼\;p\left(\sigma \right)\\ \vartheta |\vartheta_{\mathrm{pop}},\Omega \;∼\;\text{logNormal}\left(\log\;\vartheta_{\mathrm{pop}},\Omega \right),\\ \;y|c,\sigma \;∼\;\text{LogNormal}\left(\log\;c,\sigma \right). \end{array} \end{array}$$

### Specifying the model in Stan

We begin by adjusting our parameters block:

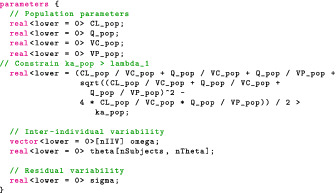

The declaration for $k_{a,\mathrm{pop}}$ illustrates that constraints may be expressions including other variables in the model. In this case, $k_{a,\mathrm{pop}}$ is constrained to avoid identifiability problems due to “flip‐flop.”

The variable, $\vartheta_{\mathrm{pop}}$ is introduced in transformed parameters, mostly for convenience purposes:

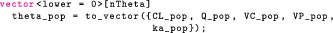

The model block reflects our statistical formulation:

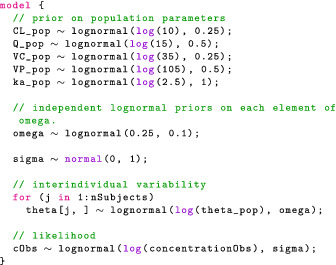

In the transformed parameters block, we also declare and calculate the individual parameters given $\vartheta_{j}$ and any relevant covariates—body weight in this case.

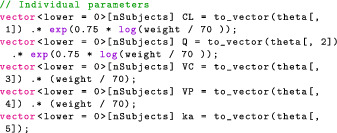

It remains to compute concentrationObs. There are several ways to do this and, depending on the computational resources available, we may either compute the concentration for each patient sequentially or in parallel. For now, we do the simpler sequential approach. In the upcoming Part 2 of this tutorial, we examine how Torsten offers easy‐to‐use parallelization for population models.

Sequentially computing the concentration is a simple matter of bookkeeping. In transformed parameters, we loop through the patients using a for loop. The code is identical to what we used in the “Transformed Data and Transformed Parameters Block” section with the caveat that the arguments to pmx_solve_twocpt are now indexed to indicate for which patient we compute the drug mass. For example, assuming the time schedule is ordered by patient, the event times corresponding to the $j^{\mathrm{th}}$ patient are given by

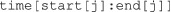

where start[j] and end[j] contain the indices of the first and last events for the $j^{\mathrm{th}}$ patient and the syntax for indexing is as in R. The full for loop is then

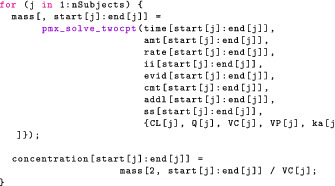

Note that the last vector argument in pmx_solve_twocpt is generated using {} syntax.

Once we have written our Stan model, we can apply the same methods for inference and diagnostics as we did in the previous section.

### Posterior predictive checks

**FIGURE 5:**
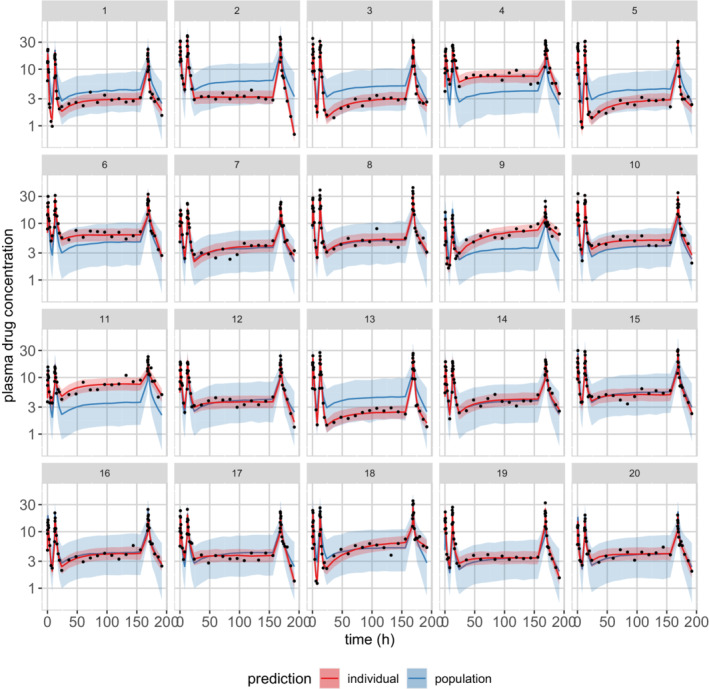
Population two‐compartment model: posterior predictive checks for each individual. Black dots = observed data, red curve and shaded area = posterior median and 90% credible intervals for the prediction of new observations in the same individual, and blue curve and shaded area = posterior median and 90% credible intervals for the prediction of new observations in a hypothetical new individual with the same body weight.

**FIGURE 6:**
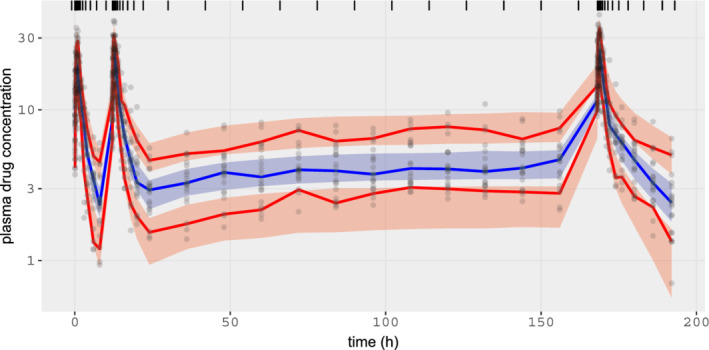
Population two‐compartment model: posterior predictive checks for all individuals. Gray circles = observed data, blue curve and shaded areas = posterior median and 80% credible intervals for the population median, and red curve and shaded area = posterior median and 80% credible intervals for the 10th and 90th population percentiles intervals.

We follow the exact same procedure as in the “Checking the Model: Posterior Predictive Checks” section—using even the same line of code—to simulate new observations for the same patients we analyzed. Figure 5 plots posterior predictions for each individual patient. In addition, we simulate new observations for hypothetical new patients by (i) drawing PK parameters from our population distribution, (ii) solving the ODEs with these simulated parameters, and (iii) using our measurement model to simulate new observations. Those predictions are also shown in Figure 5 for each individual. Figure 6 depicts a composite PPC for all individuals. The generated quantities block then looks as follows:

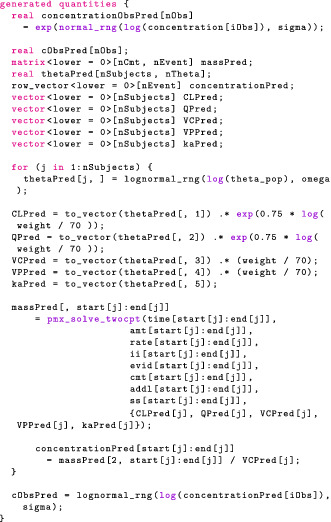

It is worth noting that the computational cost of running operations in the generated quantities is relatively small. Although these operations are executed once per iteration, in order to generate posterior samples of the generated quantities, operations in the transformed parameters and model blocks are run and differentiate multiple times per iterations, meaning they amply dominate the computation. Hence the cost of doing PPCs, even when it involves solving ODEs, is marginal. The computational scaling of Stan, notably for ODE‐based models, is discussed in the article by Grinsztajn et al.^42^

For this simple population PK modeling example with a uniform study design for all individuals, the PPCs shown in Figures 5 and 6 are arguably sufficient model diagnostics. In cases where the study design and patient populations are more heterogeneous, methods that adjust for such heterogeneity are desirable. Normalized prediction distribution errors (NPDEs)^43^ are commonly used in the maximum likelihood context and could be applied to point predictions from Bayesian models, for example, posterior mean or median predictions. A similar approach termed probability integral transforms (PIT) are used for Bayesian model checking.^25^, ^26^

Standard PPCs that use the same data for model fitting and model checking may be overoptimistic, particularly when applied to highly flexible or overparameterized models. This may be remedied by using out‐of‐sample predictions for PPCs and PITs. In the context of population models, this means fitting the model to data from a subset of individuals and predicting outcomes for the remaining individuals. This may be done for an entire data set using cross‐validation. However, generating a cross‐validation predictive distribution is computationally expensive and may often be impractical.

## NONLINEAR PK/PD MODEL

Now let us consider a PK/PD model described in terms of a nonlinear ODE system that requires the use of a numerical solver. The patient receives multiple doses at regular time intervals, and the drug plasma concentration is recorded over time.

### Nonlinear ODE model in PK/PD

**FIGURE 7:**
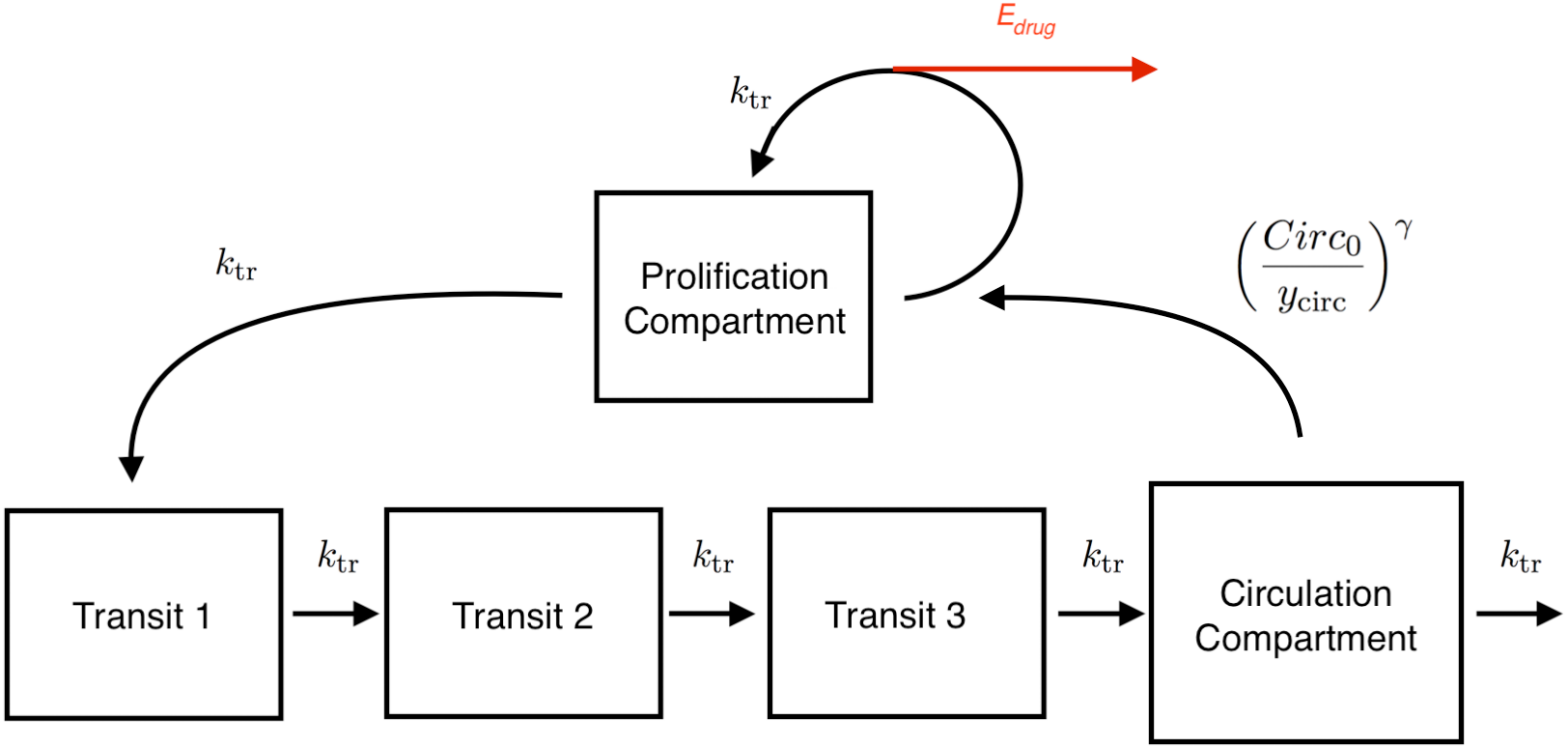
Friberg–Karlsson semimechanistic model.

In this the last example, we go back to the single‐patient, two‐compartment model and append it with a PD model. Specifically, we examine the Friberg–Karlsson semimechanistic model for drug‐induced myelosuppression^44^, ^45^, ^46^, ^47^, ^48^, ^49^ with the goal to model the relation between neutrophil counts and drug exposure. The model describes a delayed feedback mechanism that keeps the absolute neutrophil count (ANC) at the baseline (${\text{Circ}}_{0}$) in a circulatory compartment ($y_{\text{circ}}$) as well as the drug effect that perturbs this mechanism. The delay between proliferative cells ($y_{\text{prol}}$) and $y_{\text{circ}}$ is modeled by three transit compartments with mean transit time

(3) $$\mathrm{MTT}=\left(3+1\right)/k_{\mathrm{tr}}$$

where $k_{\mathrm{tr}}$ is the transit rate constant. Figure 7 summarizes the model (see also fig. 2 in Friberg et al.^44^).

The PD likelihood is

$$\begin{array}{c} \begin{array}{c} \mathrm{ANC}\;∼\;\text{logNormal}\left(\log\left(y_{\text{circ}}\right),\sigma_{\mathrm{ANC}}\right),\\ y_{\text{circ}}=f_{\mathrm{FK}}\left(\mathrm{MTT},{\text{Circ}}_{0},\alpha ,\gamma ,\hat{c}\right), \end{array} \end{array}$$

where $\hat{c}=y_{\text{cent}}/V_{\text{cent}}$ is the drug concentration calculated from the PK model, and $f_{\mathrm{FK}}$ solves the nonlinear ODE:

(4a) $$\frac{dy_{\text{prol}}}{dt}=k_{\text{prol}}y_{\text{prol}}\left(1-E_{\text{drug}}\right){\left(\frac{{\text{Circ}}_{0}}{y_{\text{circ}}}\right)}^{\gamma }-k_{\mathrm{tr}}y_{\text{prol}},$$

(4b) $$\frac{dy_{\text{trans}1}}{dt}=k_{\mathrm{tr}}y_{\text{prol}}-k_{\mathrm{tr}}y_{\text{trans}1},$$

(4c) $$\frac{dy_{\text{trans}2}}{dt}=k_{\mathrm{tr}}y_{\text{trans}1}-k_{\mathrm{tr}}y_{\text{trans}2},$$

(4d) $$\frac{dy_{\text{trans}3}}{dt}=k_{\mathrm{tr}}y_{\text{trans}2}-k_{\mathrm{tr}}y_{\text{trans}3},$$

(4e) $$\frac{dy_{\text{circ}}}{dt}=k_{\mathrm{tr}}y_{\text{trans}3}-k_{\mathrm{tr}}y_{\text{circ}},$$

We use

$$E_{\text{drug}}=\min\left(\alpha \hat{c},1\right)$$

to model the linear effect of the drug once it has been absorbed in the central compartment. This effect reduces the proliferation rate and induces a reduction in neutrophil count. The upper bound of 1 on $E_{\text{drug}}$ excludes the scenario where the feedback loop is flipped if $\hat{c}$ becomes too large. Although we expect that for any reasonable parameter values, $E_{\text{drug}}<1$, we should anticipate the possibility that our Markov chains may encounter less well‐behaved values as it explores the parameter space. Encoding such constraints can lead to improved numerical stability when solving the ODE.

We obtain the complete ODE system for the PK/PD model by coupling Equations (1) and (4). Because the equation is nonlinear, we can no longer resort to analytical solutions as we have done in the previous sections.

#### Numerically solving ODEs

To solve an ODE numerically in Stan we first need to define a function that returns a right‐hand side of the ODE, that is, the derivative of the solution, in the functions block. The functions block allows users to define functions and is written at the top of the Stan file before the data block.

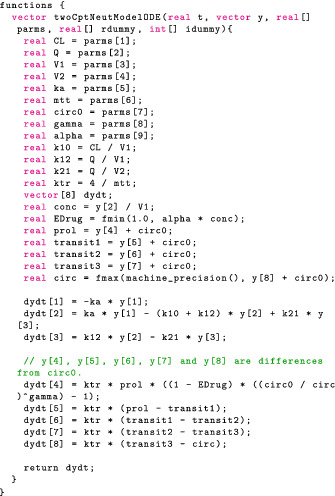

This function is an almost direct translation of Equations (1) and (4). The first three components of dydt describe the PK. The next five components of dydt describe the PD minus the baseline ${\text{Circ}}_{0}$. Writing the ODE as a difference from the baseline means the initial PD conditions is 0, as opposed to a parameter dependent value. This results in better computation because derivatives of the ODE solution with respect to the initial conditions no longer need to be computed; for more details, see section 5.2 in Grinsztajn et al.^42^ In addition, we encode a constraint on the circulatory compartment

$$y_{\text{circ}}>\varepsilon >0,$$

where ε is the machine precision and can be interpreted as the smallest nonzero number the computer can handle. This is to improve numerical stability, especially during the early stages of MCMC exploration when we may need to handle somewhat implausible parameter values.

Stan and Torsten provide several numerical solvers. In this example, we use the Runge–Kutta solver pmx_solve_rk45 (section 3.4 in Zhang et al.^33^). The signature of pmx_solve_rk45 is a bit more sophisticated than that of pmx_solve_twocpt and requires the following arguments:

1. the name of the user‐defined ODE function (twoCptNeutModelODE)
2. the number of states/compartments in the ODE
3. the event schedule
4. the bioavailability fraction, F, and the dosing lag time, $t_{\mathrm{lag}}$ for each compartment (optional)
5. the tuning parameters for the ODE solver (optional)

Because arguments are nameless in Stan, we can only pass the ODE tuning parameters if we also pass F and $t_{\mathrm{lag}}$. By setting F to 1 and $t_{\mathrm{lag}}$ to 0 for each compartment, we essentially ignore their effect. This is best done in the transformed data block:

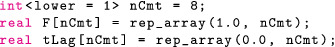

Numerical solvers in Stan and Torsten admit three tuning parameters:

• rtol: relative tolerance to determine solution convergence,

• atol: absolute tolerance to determine solution convergence,

• max_num_step: maximum number of steps allowed. Although Stan and Torsten provide default values, we highly recommend that the user define the ODE solver control parameters in the data block:

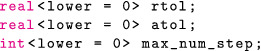

Users should make problem‐dependent decisions on rtol and atol, according to the expected scale of the unknowns, so that the error does not affect our inference. For example, when an unknown can be neglected below a certain threshold without affecting the rest of the dynamic system, setting atol greater than that threshold avoids spurious and error‐prone computation. For more details, see Chapter 13 in the Stan User's Guide^37^ and section 3.7.5 in Zhang et al.^33^ and references therein.

As before, we solve the ODE within the event schedule in the transformed parameters block:

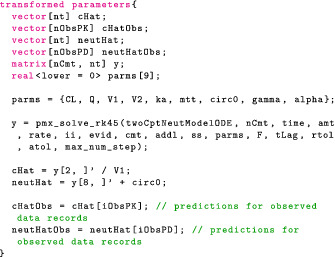

#### Solving PK/PD ODEs as a coupled system

The approach in the last section applies to all models that involve ODE solutions, but we will not use it here. An acute observer may have noticed the PK/PD model here exhibits a particular *one‐way coupling* structure. That is, the PK (Equation 1) and PD (Equation 4) are coupled through the proliferation cell count $y_{\text{prol}}$ and $E_{\text{drug}}$, such that the PK can be solved independently from the PD. This is what motivates Torsten's coupled solvers that analytically solve the PK ODEs before passing the PK solution to the PD ODE. The PD ODE is then solved numerically. Because the dimension of the numerical ODE solution is reduced, in general this coupled strategy is more efficient than the last section's approach of numerically solving a full ODE system. To see it in action, let us apply the coupled solver pmx_solve_twocpt_rk45 (section 3.5 in Zhang et al.^33^) to the same model. We need only make two changes. First, we modify the ODE function to reflect that only the PD states are to be solved.

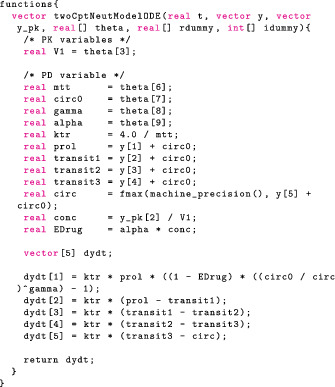

Note that we pass in PD and PK states as separate arguments, y and $y_{\mathrm{PK}}$, respectively. The above function only returns dy/dt, while $y_{\mathrm{PK}}$ is solved internally using an analytical solution, meaning users do not need to explicitly call pmx_solve_twocpt.

Then we replace pmx_solve_rk45 with pmx_solve_twocpt_rk45 call.

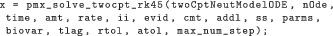

### Building the remaining coding blocks

We omit the data block but note that it is similar to the one we constructed in previous sections. The key difference is we now include measurements for the absolute neutrophil count. The parameters block now contains the PD variables:

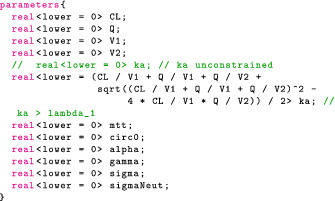

The model block is similar to that in “PK Model and Clinical Event Schedule” section:

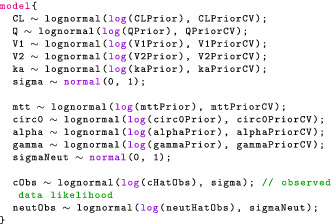

#### Posterior predictive checks

We hope by now the reader has developed the habit of performing PPCs on every model. Because we have both PK (drug concentration) and PD (neutrophil count) observations, the PPC should be conducted on both.

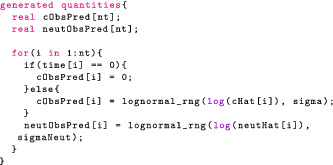

**FIGURE 8:**
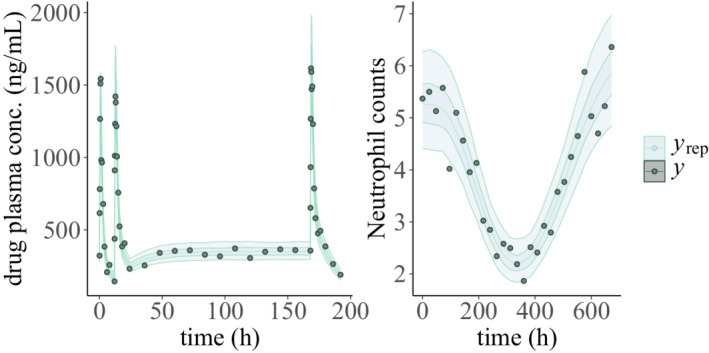
Posterior predictive checks for the pharmacokinetic/pharmacodynamic model. The circles represent the observed data (y) and the shaded areas the 50th and 90th credible intervals based on posterior draws ($y_{rep}$)

It is possible to only run the generated quantities block based on a fitted model using cmdstanr's generate_quantities routine. This is useful when we change the generated quantities, but not the rest of a model we have already fitted. The compiled model and the fit are respectively stored in the mod and fit objects in R. We then run:

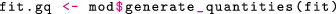

and use the results for a PPC (Figure 8).

## DISCUSSION

Stan provides an expressive language to build models, state‐of‐the‐art algorithms to fit these models, and a host of easy‐to‐use diagnostics. Torsten complements Stan with a suite of routines that solve ODEs within the context of clinical event schedules. Together, Stan and Torsten are potent tools when working through the tangled steps of a Bayesian workflow for PK/PD modeling.

### Current and potential role for Stan and Torsten for pharmacometrics applications

We can apply Stan/Torsten to a large palette of generative models, both for inference and simulation. Applications range from simple linear regression to complex multiscale quantitative systems pharmacology models. Compared with specialized pharmacometrics tools such as NONMEM®, Stan/Torsten is particularly well suited for cases where more flexibility is desired. This includes models with

• random‐effects distributions other than normal,

• prior distributions other than the limited set available in existing pharmacometrics tools,

• multiple submodels with different random‐effect structures.

It is important to recognize that MCMC, including the HMC scheme used by Stan/Torsten, can be computationally intensive, notably when fitting hierarchical models that require us to numerically solve ODEs. This can be especially frustrating during the initial model exploration stage of a project. For such exploratory analyses, access to a rapid approximate Bayesian inference engine may be desirable. Stan/Torsten includes two optimization‐based inference engines, one for estimation of posterior modes and one for variational inference. These algorithms attempt to simultaneously optimize over the entire joint posterior distribution of all model parameters. This process can be relatively slow and error prone when trying to optimize over the large number of population and individual‐level parameters of a typical population pharmacometrics model. This contrasts with typical mixed‐effects modeling programs that use algorithms specialized for a more limited range of models—usually employing an alternating sequence of lower dimensional optimization problems.

For applications that may be implemented with typical pharmacometrics tools, the choice between those and Stan/Torsten comes down to the trade‐offs between flexibility, doing accurate Bayesian inference, and computation time.

We would also like to point out that Stan is not the only probabilistic programing language that is actively under development. PyMC3,^50^ TensorFlow Probability,^51^, ^52^ and Turing,^53^ among others, provide similar modeling capabilities. A full review and comparison of these languages is, however, beyond the scope of this article.

### Preview of Part 2

In Part 2 of this tutorial, we plan to build on the material we have covered thus far and tackle more advanced topics, including:

• *Improving the performance of HMC*, using within‐chain parallelization for population models and Torsten's dedicated group solvers.

• *Advanced diagnostic tools*, namely, divergent transitions that can flag bias in our posterior samples. Stan makes these diagnostics readily available.

• *Fake data simulation and analysis,* in particular prior predictive checks as a way to understand and build priors, fitting the model to fake data as an imperfect tool to troubleshoot Bayesian inference, and an overview of the more sophisticated but computationally demanding simulation‐based calibration.^54^

• *Performance tuning of ODE models*, such as solver selection and accuracy control as well as stability issues.We will dive into these subjects by examining more advanced models and using techniques such as reparameterization, within‐chain parallelization, and pooling multiple data sources. We will also discuss ongoing developments with Stan and Torsten, such as tools to handle larger scale ODEs and plans to leverage parallelization.

## CONFLICT OF INTEREST

The authors declared no competing interests for this work.
